# Supply factors as determinants of treatment costs: clinicians’ assessments of a given set of referrals to community mental health centers in Norway

**DOI:** 10.1186/s12913-018-2884-5

**Published:** 2018-01-30

**Authors:** Knut Reidar Wangen, Sverre Grepperud

**Affiliations:** Department of Health Management and Health Economics, University of Oslo, P.O. box 1089, Blindern, 0318 Oslo, Norway

**Keywords:** Mental health care utilization, Length of stay, Outpatient services, Supply factors

## Abstract

**Background:**

Previous works that uses patterns of prior spending to predict future mental health care expenses (utilization models) are mainly concerned with demand (need) variables. In this paper, we introduce supply variables, both individual rater variables and center variables. The aim is to assess these variables’ explanatory power, and to investigate whether not accounting for such variables could create biased estimates for the effects of need variables.

**Methods:**

We employed an observational study design where the same set of referrals was assessed by a sample of clinicians, thus creating data with a panel structure being particularly relevant for analyzing supply factors. The referrals were obtained from Norwegian Community Mental Health Centers (outpatient services), and the clinicians assessed the referrals with respect to recommended treatment costs and health status.

**Results:**

Supply variables accounted for more than 10% of the total variation and about one third of the explained variation. Two groups of supply variables, individual rater variables and center variables (institutions) were equally important.

**Conclusions:**

Our results confirm that supply factors are important but ignoring such variables, when analyzing demand variables, do not generally seem to produce biased (confounded) coefficients.

**Electronic supplementary material:**

The online version of this article (10.1186/s12913-018-2884-5) contains supplementary material, which is available to authorized users.

## Background

Studies that explain the use of mental health care resources (utilization rates and treatment costs) are key components of any attempt to describe and account for the operation of psychiatric services, and the identification of modifiable determinants could lead to improvements in the quality of care [1]. Information on variables explaining treatment cost can also be used; (i) for planning purposes (e.g. identifying cost drivers), (ii) for risk- adjustment (resource allocation formulae), (iii) for the design of activity-based reimbursement systems, and, (iv) as inputs into cost - and economic evaluation analyses.

There is a substantial body of literature into the factors explaining variation in mental health care resources. Most studies are concerned with adult psychiatric inpatients and some date back almost three decades when the interest into the use of diagnosis-related groups (DRGs) sparked [2–8]. The dominant perspective is a demand perspective where the independent need variables are expected to be associated with mental health problems including patient characteristics (risk factors) such as clinical variables (e.g., diagnosis, co-morbidities, prior treatment history, severity of illness indicators), demographic variables (e.g., age, gender, ethnicity), and deprivation variables (e.g., employment, income).

In this article, we are exploring the role of supply variables in explaining variation in mental health treatment costs. We are concerned with; (i) the share of total variance and the share of the explained variation, (ii) the relative importance of types of supply variables (center- and rater effects), (iii) the significance of supply variables relative to demand variables, and, (iv) to what extent demand and supply variables are confounded. It should be noted that the concepts of demand and supply variables, as applied here, do not reflect a belief in the two groups of variables being separated. For example, assessments along clinical dimensions such as health status and diagnosis are typically performed by agents on the supply-side of health care markets (clinicians) thus causing an interdependency between the two groups of variables. Our analyses are based on data for outpatients referred to Community Mental Health Centers (CMHCs) in Norway. The Norwegian health care system resembles that of Great Britain and other Scandinavian countries with mainly public ownership and funding through taxes. The responsibility for specialist care is delegated to 4 Regional Health Authorities (RHAs), while primary care responsibility lies with the municipalities [9]. The Norwegian CMHCs are relatively homogenous in several respects since being publicly owned and publicly funded (mainly fixed global budgets) with a catchment area responsibility.

A general finding from the literature on mental health care utilization is that clinical variables have better explanatory power than demographic variables [10], while the effects from diagnosis (disorders) are found to depend on the type of classification system used [11, 12]. Diagnosis Related Groups (DRGs), for example, are not doing particularly well, while other classification systems do somewhat better [13, 14]. Furthermore, psychosis, compared with non-psychotic categories, is frequently associated with an increased length of stay [1]. Other variables reported as important are age, comorbidity (drugs and alcohol) and previous admissions [1, 11, 12, 15, 16]. Hermann et al. (2007), in a survey on mental health and substance–related care that consider 72 models on utilization and 74 models on expenditures, found that models based upon diagnostic and socio-demographic information from administrative data sets explain on average only 6.7% of the total variance while more detailed sources of data on average explain 22.8% [17].

Supply variables (sometimes referred to as “non-need” variables) that may explain variation in utilization rates include institutional (center) variables and rater variables (clinician- or practice pattern variables). Institutional (center) factors that might influence length of stay include resource availability, payment structures, production efficiency, cost structure (economics of scale, slack and ownership) and location (physical presence). Variation attributable to the policies and practices of the treating clinician(s) (practice pattern) might be provider preferences, theoretical orientation, treatment goals, and the use of specific preferred modalities. In universal health care systems where equal access concerns are important, an important objective is that supply (non-need) variables do not explain treatment cost variation.

A general concern with utilization models focusing on need variables (demand) is that of individuals using services only when they have access to care which again also may depend on both institutional variables as well as individual rater variables. Ignoring supply variables may cause biased estimates (confounded estimators) if demand and supply variables are correlated. This again may sustain existing inequity (both horizontal and vertical) in the provision and planning of health care, for example by “over-rewarding” providers already being well supplied. Ignoring supply variables may also be one reason for why demand-oriented studies tend to explain a relatively low degree of the overall variation in resource use.

The literature that address supply factors is mainly concerned with institutional variables. A first group of studies is studying the effect from different payment methods (reimbursement structures) on resource use (utilization). Rupp et al. (1985) introduce hospital fixed effects to capture location and teaching status [3]. Frank and Lave (1986) control for economics of scale and slack (X-efficiency) by including hospital size and ownership (public or non-public) [4]. Lave and Frank (1990) include hospital bed size and teaching intensity [18]. A second group of studies, using more aggregated data, estimates formulae used to distribute health care resources between areas or regions (resource allocation formulae) [19]. These studies typically introduce regional dummy variables to control for differences in supply [20, 21]. In addition, there are also two single center studies that consider the role of practice patterns on the use of mental health care services. Lyons et al. (1991) find, from introducing physician-specific fixed effects, that about 10% of the overall variation in length of stay is attributable to variation in practice patterns [22]. Huntley el al. (1998) estimates a demand regression model and compares actual mean length of stay with predicted mean length of stay for each physician [23]. They find that the behavior of individual physicians is not a significant predictor.

The main finding of our multi-center study, including both institutional and individual rater variables, is that treatment costs vary significantly across clinicians and that this variation is explained by both demand and supply variables. Second, the demand variables (need variables), considered as a group, are somewhat more important than the supply variables. Third, the supply variables play an important role, and, considered as a group, such factors are responsible for about a third of the explained variation in treatment costs and about 10 % of the total variation. Fourth, the two types of supply variables included in our analysis, practice patterns and center effects, are equally important. Fifth, we find no support for the omittance of supply variables to produce biased estimates. Our work contributes to the established literature by studying outpatients and by analyzing supply variables when demand variables are fully controlled for. This is made possible because our multi-center data include several clinicians’ ratings of identical referrals.

## Methods

Based upon former findings from the literature, outpatient mental health care treatment costs at CMHCs are hypothesized to depend on demand variables (patient characteristics) and supply variables (individual rater variables, i.e. practice patterns, and variables at the institutional level). Our aim is to explore the explanatory power of these groups of variables.

We employed an observational study design, where the data set is generated from a letter distributed to all clinicians (69 in total) being involved with admission work (assessing referrals) at the CMHCs in the South-East Health Region of Norway during April and May 2009 (34 in total), covering alone about 58% of the Norwegian population. The letter contained 20 anonymized referrals (case vignettes) from General practitioners (GPs), one assessment form, and a questionnaire. Although being anonymized, the referrals would reveal information on gender and presumably approximate age, depending on the informational content of each letter. This research project, however, did not have access to any patient background variables. The 20 referrals were selected from a collection of 600 anonymized referrals submitted to five CMHCs during 2008 and were to reflect variation in symptoms, health state, and diagnosis (type of disorders). More details on the selection of vignettes are available from Grepperud et al. (2014) [24]. The 41 clinicians who reported treatment costs (a response rate equal to 60%) belonged to 14 different CMHCs.

The clinicians assessed the patients based on referral letters. The resulting data had a panel structure since all participating clinicians assessed each referral. This again produced data being particularly relevant for analyzing supply factors, since, for each referral, demand factors (patient characteristics) can be controlled for. The same data allows us to introduce referral (patient)-specific effects that can be said to represent a very detailed diagnosis classification system.

The main variable of interest was treatment costs. Each clinician was asked to suggest treatment profiles by choosing among the three treatment types currently being supplied: (i) consultations, (ii) day-patient care, and, (iii) in-patient treatment. For every chosen treatment type, a treatment intensity recommendation was to be reported. By combining the recommended treatment profiles with unit costs, we arrived at a treatment cost estimate for each rater for every referral. The unit costs were calculated based on financial accounts and personal communication with accounting managers at some CMHCs: (i) 1500 NOK per consultation, (ii) 1000 NOK per day, and, (iii) 3.500 NOK per night (1 Euro = 8.80 Norwegian kroner (NOK)).

In their assessments, clinicians rated the patients by using the dual version of The Global Assessment Scales (GAF). This instrument is described, discussed and analyzed in many publications and is intended to be generic rather than a diagnosis-specific system [25–28]. The dual version of GAF is routinely used by Norwegian CMHCs in clinical practice and has separate scores (0-100) for symptoms (GAF-S) and functioning (GAF-F). We defined GAFmin as the lowest (most severe) score of GAF-S and GAF-F. The clinicians also assessed whether or not each patient had a psychotic disorder, a substance abuse problem, and/or was suicidal. Finally, the clinicians were asked to answer a questionnaire including questions on profession, education, rater experience and whether being a manager or not.

Several methods for modeling costs have been debated. In our case, there are three main alternatives: ordinary least square (OLS) regressions for either costs or log-transformed costs, or generalized linear models with a log-link function and a gamma distribution. Previous comparisons of these methods suggest that their results may differ but there is no perfect method for the analysis of mental health cost data [29].

Our aim was to compare goodness of fit and the coefficients of key explanatory variables, across several models with different sets of regressors. We chose to use OLS for log-transformed costs, and to use adjusted R-squared to measure goodness of fit. All estimated models are variants of the following equation for the log of treatment costs, *C*_*ij*_, for referral *i* and clinician *j*:

$$ {C}_{ij}=\alpha +\beta {D}_{ij}+{v}_i+\gamma {S}_j+\delta {u}_{ij}+{r}_j+{\varepsilon}_{ij}.\kern2.25em (1) $$

The full version of this equation was not estimated, and the models differ in respect to which parts of the equation that were excluded. For instance, Model II can be written *C*_*ij*_ = *α* + *βD*_*ij*_ + *γS*_*j*_ + *ε*_*ij*_, where α is a constant, *D*_*ij*_ is a vector of clinical variables, *S*_*j*_ is a vector of rater variables, and ε_*ij*_ is an error term. These variables are presented and described in Table 1. Some models also included referral-specific fixed effects (*v*_*i*_), center-specific fixed effects (*u*_*ij*_), or rater-specific fixed effects (*r*_*j*_).

**Table 1:** Variables and descriptive statistics for sample used in Models I, II, III and IV (*n* = 523)^a^

	Variables	Mean (proportion)	Std. Dev.	Min	Max
	Treatment costs	133,802	116,348.3	1500	742,500
	Ln(Treatment costs)	11.50	0.80	7.31	13.52
Clinical variables (D_ij_)	GAFmin	44.3	10.3	10	72
	Psychosis: (=1 if symptoms of psychosis; if not = 0)	(0.28)			
	Suicidal: (=1 if symptoms of suicidality; if not =0)	(0.40)			
	Substance abuse: (=1 if having a substance abuse problem; if not =0)	(0.30)			
Rater background variables (S_j_)	Profession				
	Psychiatrist: (if psychiatrist =1; if not =0)^b^	(0.36)			
	Psychologist:(if psychologist =1; if not =0)^b^	(0.37)			
	Education: (if specialist = 1; if not =0)	(0.88)			
	Manager: (if manager =1; if not =0)	(0.64)			
	Rater experience: (if more than two years of experince = 1; if two years or less = 0).	(0.61)			

^a^Some corresponding descriptive statistics for the full sample are available in Grepperud et al. [24]

^b^In addition to being a psychiatrist or a psychologist, the respondents could belong to professions such as nurses and social workers

Patient characteristics can be modelled quite flexibly by referral-specific effects (*v*_*i*_*)* while for rater characteristics the same matters for center-specific effects (*u*_*ij*_*)* and rater-specific effects (*r*_*j*_). *D*_*ij*_ and *S*_*j*_*,* on the other hand*,* include variables better suited for interpretation and generalization.

The distribution of log-costs did not appear to have deviations from normality sufficient to invalidate the statistical inference, confer the Additional file 1: Figure S1. In the estimations, we calculated robust standard errors (Huber and White, or sandwich estimator) to allow for potential heteroscedasticity. We also ran non-parametric bootstrap simulations, which gave rather similar standard errors.

## Results

Table 1 presents descriptive statistics for the samples used in Models I-IV. Table 2 presents information on treatment costs for each referral including the number of assessments *(N*), means, quartiles, and the coefficients of variation, while Tables 3 and 4 present information on the rater assessments of the clinical variables. Tables 5 and 6 present the results of the regressions. In total, we present seven empirical models. The three models of Table 6 include fixed effects only, while the four models presented in Table 5 also include demand and supply variables.

**Table 2:** The distribution of treatment costs across referrals in Norwegian kroner (NOK). € 1 = NOK 8.80

Referral	N^a^	Mean	Median	Lower quartile (25%)	Upper quartile (75%)	Max/Min	Coefficient of variation
1	25	123,900	72,000	60,000	156,000	25.0	1.00
2	27	123,444	120,000	72,000	156,000	20.8	0.59
3	33	156,854	147,000	49,000	180,000	25.5	0.85
4	35	257,529	196,000	144,000	312,000	15.2	0.68
5	6	59,000	51,000	24,000	60,000	6.0	0.75
6	29	86,466	72,000	49,000	120,000	156.0	0.61
7	37	85,622	72,000	48,000	96,000	4.3	0.45
8	14	68,357	66,000	30,000	78,000	13.3	0.65
9	27	73,556	60,000	36,000	96,000	13.0	0.57
10	16	34,688	27,000	18,000	48,000	8.0	0.63
11	35	141,586	103,500	54,000	156,000	42.0	0.94
12	28	81,321	72,000	51,000	108,000	13.0	0.51
13	35	292,429	294,000	156,000	312,000	14.2	0.58
14	26	123,519	120,000	72,000	156,000	13.0	0.61
15	32	108,984	96,000	72,000	156,000	6.0	0.41
16	38	143,895	121,250	72,000	225,000	13.0	0.63
17	32	151,531	156,000	72,000	215,000	13.0	0.60
18	35	90,729	72,000	45,000	120,000	16.3	0.77
19	37	87,000	72,000	60,000	120,000	6.5	0.44
20	28	110,893	108,000	60,000	156,000	13.3	0.58
Sum	575	2,412,313	2,097,750	1,244,000	3,026,000	744.3	
Mean (all referrals)		120,615	104,887	62,200	151,300	37.2	

^a^N = number of assessments

**Table 3:** The distribution of Global Assesment Scale scores (GAFmin) across referrals

Referral	N^a^	Mean	Median	Lower quartile (25%)	Upper quartile (75%)	Max/Min	Coefficient of variation
1	24	53.3	53.5	47.5	60.0	2.0	0.16
2	26	49.9	50.0	48.0	55.0	2.0	0.13
3	31	37.1	40.0	31.0	43.0	5.0	0.24
4	32	32.2	32.5	26.5	40.0	4.8	0.27
5	5	63.0	60.0	60.0	65.0	1.2	0.07
6	26	46.9	48.0	42.0	50.0	1.7	0.13
7	33	53.1	53.0	50.0	55.0	1.4	0.08
8	13	43.5	42.0	40.0	48.0	1.4	0.11
9	25	48.5	50.0	45.0	52.0	1.5	0.11
10	15	44.5	45.0	41.0	47.0	1.6	0.11
11	32	44.2	45.0	40.0	47.0	1.5	0.10
12	27	51.2	50.0	48.0	55.0	1.5	0.09
13	32	27.3	28.0	25.0	30.0	1.9	0.17
14	23	52.8	52.0	48.0	55.0	1.8	0.16
15	25	40.8	43.0	35.0	45.0	2.8	0.22
16	33	34.9	35.0	30.0	40.0	2.5	0.21
17	29	38.4	39.0	35.0	40.0	3.2	0.20
18	33	53.2	53.0	50.0	55.0	1.9	0.12
19	34	46.5	47.0	45.0	50.0	1.6	0.10
20	25	46.3	47.0	42.0	50.0	1.7	0.11

^a^N = number of assessments. The total number of assessments = 523

**Table 4:** Assessments of whether a patient had a psychotic disorder, was sucidal, or had a substance abuse problem^a^

Referral	N	Psychosis		Suicidal		Substance abuse	
		Mean	(n)	Mean	(n)	Mean	(n)
1	24	0.00	(0)	0.04	(1)	1.00	(24)
2	26	0.19	(5)	0.08	(2)	1.00	(26)
3	31	0.03	(1)	1.00	(31)	0.00	(0)
4	32	0.97	(31)	1.00	(32)	0.25	(8)
5	5	0.00	(0)	0.00	(0)	0.00	(0)
6	26	0.00	(0)	0.88	(23)	0.00	(0)
7	33	0.00	(0)	0.03	(1)	0.00	(0)
8	13	0.00	(0)	0.62	(8)	0.54	(7)
9	25	0.00	(0)	0.00	(0)	0.08	(2)
10	15	0.00	(0)	0.00	(0)	0.93	(14)
11	32	0.19	(6)	0.59	(19)	0.03	(1)
12	27	0.00	(0)	0.04	(1)	0.07	(2)
13	32	0.97	(31)	0.41	(13)	0.13	(4)
14	23	0.04	(1)	0.00	(0)	0.61	(14)
15	25	0.28	(7)	1.00	(25)	0.24	(6)
16	33	1.00	(33)	0.97	(32)	0.21	(7)
17	29	1.00	(29)	0.10	(3)	0.14	(4)
18	33	0.09	(3)	0.00	(0)	1.00	(33)
19	34	0.00	(0)	0.38	(13)	0.09	(3)
20	25	0.00	(0)	0.20	(5)	0.04	(1)

^a^N = number of assessments; n = number of positive assesments (e.g., Psychosis = 1); Mean = proportion of positive assessments. The total number of assesments = 523

**Table 5:** Multivariate regression models for the log of costs. Coefficients and robust standard errors^a^

	Model I			Model II			Model III^b^			Model IV ^b^		
	Coeff	SE	p	Coeff	SE	p	Coeff	SE	p	Coeff	SE	p
Constant term	**11.673**	0.214	< 0.001	**12.010**	0.234	< 0.001	**12.138**	0.282	< 0.001	**11.976**	0.310	< 0.001
GAFmin	**−0.008**	0.004	0.056	**− 0.011**	0.004	0.008	**−0.014**	0.004	0.001	**−0.010**	0.005	0.063
Psychosis	**0.551**	0.091	< 0.001	**0.525**	0.089	< 0.001	**0.500**	0.088	< 0.001	**0.268**	0.161	0.097
Suicidal	**0.129**	0.070	0.068	0.083	0.070	0.236	0.032	0.069	0.643	0.004	0.132	0.974
Substance abuse	−0.067	0.070	0.341	−0.040	0.070	0.569	− 0.041	0.067	0.542	−0.034	0.091	0.707
Psychiatrist				**−0.248**	0.083	0.003	**−0.194**	0.101	0.055	**−0.164**	0.091	0.074
Psychologist				**−0.158**	0.079	0.047	−0.073	0.108	0.501	−0.033	0.099	0.740
Education				**0.243**	0.098	0.014	0.186	0.143	0.196	0.160	0.132	0.225
Manager				**−0.172**	0.066	0.009	**−0.193**	0.113	0.087	**−0.183**	0.103	0.076
Rater experience				**−0.232**	0.066	0.001	−0.111	0.079	0.161	**−0.140**	0.072	0.054
Referral-specific spespecific fixed effects:												
Average										−0.015		
Std.dev										0.381		
Center-specific fixed effects:												
Average							−0.013			−0.033		
Std.dev							0.280			0.289		
Adj R-sq	0.171			0.213			0.276			0.383		

^a^N = 523 in models I-IV. Coefficients with two-sided *p*-values less than 10% are in boldface

^b^For brevity only the averages and standard deviations of the estimated fixed effects are reported in this table; all estimated coefficients are presented in the Additional file 1: Table S2

**Table 6:** Multivariate fixed effect regression models for the log of costs^a^

	Model V	Model VI	Model VII
Referral-specific fixed effects:			
Average	−0.008		0.041
Std.dev	0.487		0.480
Rater-specific fixed effects:			
Average		−0.023	−0.024
Std.dev		0.344	0.338
Adj R-sq	0.256	0.123	0.391

^a^N = 575 for models V-VII. For brevity only the averages and standard deviations of the estimated fixed effects are reported in this table; all estimated coefficients are presented in the Additional file 1: Table S3

Table 1 shows that the average treatment costs were 133,802 NOK. Overall, consultations accounted for 80.1% of the treatment costs, while day-care and inpatient stays accounted for 1.1% and 18.8%, respectively.

Table 2 shows that the number of observations varied substantially between the referrals because of missing observations. There is evidence of a substantial within-referral variation. First, it is observed that the average mean treatment costs for the referrals are about NOK 120,000 (€13,600). Second, the mean treatment costs (and the median) vary substantially across the 20 referrals e.g., referral 13 is 8.5 times more expensive than referral 10. By comparing means and medians, it follows that for all referrals, with the exception of 13 and 17, the distributions are skewed to the right. Measured by the coefficient of variation (the standard deviation divided by the mean), referrals 1, 3 and 11 have the most pronounced variation, while the variation is lowest for referrals 7, 15, and 19. The ratio between the maximum and minimum values is highest for referral 6 and lowest for 7. The sum of treatment costs for the maximum values across all twenty referrals (6.59 million NOK) is about 14.5 times higher than the sum of treatment cost if using the minimum values (0.45 million NOK). In sum, the above observations suggest a strong disagreement among clinicians with respect to treatment costs for similar patients (having identical patient information). Further information on the treatment types recommended for the referals is reported in the Additional file 1: Table S2.

Tables 3 and 4 show that also the assessment of clinical variables varied noticeably between referrals and between raters (i.e., within referrals). For referral 19, 13 out of 34 raters (38%) assessed the patient to be suicidal, while the other raters did not (Table 4). Only referral 5 obtained unanimity regarding the three dichotomous clinical variables in Table 4 (Psychosis = Suicidal = Substance abuse = 0) and this was a relatively mild case (high GAFmin, Table 3) which most clinicians deemed unnecessary to treat [24]. Clinicians appear to agree more regarding the Psychosis variable than they do for the Suicidal and Substance abuse variables: In Table 4, 12 referrals had mean values equal to 0.00 or 1.00 (i.e., unanimity) for Psychosis, compared to respectively 8 and 7 referrals for Suicidal and Substance abuse. Moreover, only one referral (referral 15) had a mean value between 26% and 74%, compared to four referrals for Psychosis (referrals 8, 11, 13, and 19) and two referrals for Substance abuse (referrals 8 and 14).

Table 5 presents results for four regression models, Models I, II, III and IV. Model I is the simplest model including clinical variables only. Models II, III and IV successively add variables for rater characteristics, center-specific fixed effects and referral-specific effects. In our discussion, we consider a coefficient significant if its two-sided *p*-value is 10% or lower.

The coefficient of GAFmin measures approximately the percentage change in costs when GAFmin increases by one point, ceteris paribus. In Model I the estimated value is − 0.012, which suggests that such a change would reduce costs by 1.2%. The estimated coefficients for GAFmin, ranging between − 0.10 and − 0.015, are significant in all models. Psychosis is also significant in all four models. The coefficient of Psychosis measures the change in log-costs when comparing patients diagnosed with psychosis to otherwise similar patients without psychosis. The corresponding difference in costs is obtained by taking the anti-log, which for Model I yields *exp.*(0.551) = 1.735, and the interpretation is that patients with psychosis cost 73.5% more than patients without psychosis do. Substance abuse is not significant in either of the models while Suicidal is significant in Model I only.

Practice pattern factors are represented by rater characteristics in Models II–IV. In all three models, both variables Psychiatrist and Manager are significant with negative coefficients, implying that psychiatrists, relative to non- psychiatrists, and managers, relative to non-managers, tend to recommend lower treatment costs. Estimates for differences in costs can again be obtained by taking the anti-log. For Manager in Model II, we have *exp.*(− 0.173) = 0.841, implying that managers recommend costs that are (1-0.841 = 0.159) 15.9% below the non-managers recommendations. Rater experience has negative coefficients that are significant in Models II and IV, but not significant in Model III. The variables Psychologist and Education have significant coefficients in Model II only.

Table 5 reports the average and standard deviation for the estimated center-specific effects, while the individual estimates can be found in the Additional file 1: Table S2. The numeric values of the averages, − 0.013 and − 0.033 in Models III and IV, respectively, are small by construction (confer notes to the Additional file 1: Tables S2 and S3) and can be viewed as simple adjustments to the constant terms. The standard deviations, 0.280 and 0.289, are more relevant for interpretation: In Model IV, suppose we compare the average center with a center whose center-specific effect is one standard deviation above the average. Then the estimated mean difference in log-costs will be 0.289, corresponding to a 33.5% difference in treatment costs [*exp*(0.289) = 1.335].

In order to control for unobserved variables we estimated regressions that only included referral-specific effects and/or rater-specific effects, confer Table 6. Model VII includes both referral and rater-specific effects and explains 39.1% of total variation, which is slightly higher than Model IV (38.3%). Model V includes only referral-specific effects and explains 25.6%, notably higher than Model I (16.6%).

## Discussion

Information on factors explaining mental health care utilization is important for planning purposes. Most existing studies identifying such factors study inpatients (hospitals) with a demand (need) perspective. Those studies that do include supply variables normally do so by including hospital characteristics such as size and ownership. In addition, a few studies investigate the potential role of the practice patterns of individual clinicians. In our study, we include both organizational- and practice pattern variables. Moreover, since the data contain several observations for each referral, and the clinicians are nested within centers, we can control for fixed demand and supply factors.

Our estimates of demand effects (need variables) are consistent with several former studies. Lower admission GAF scores have been found to be associated with longer length of stay [5, 30], and psychosis have been reported as an important factor explaining treatment costs [3, 10].

We do not know of other multi-center studies that includes provider variables (practice pattern factors) when analyzing the utilization of mental health care services. However, the effect found for Manager is not surprising since having an overall budgetary responsibility can be expected to reduce recommended treatment intensities.

The center-specific fixed-effects included in models III and IV can be seen as a way to control for factors that are not accounted for or are unobservable, such as size, resource availability, culture and organization. In our study, the center-specific effects will not reflect differences in ownership or reimbursement system, since all participating centers are publicly owned and financed. A few former studies on mental health care utilization include institutional variables, and hospital size has been found to be associated with length of stay [4, 6, 18]. Hospital-specific effects have been introduced in modelling but not reported [3].

Thus, it is worth noting that the results for the demand variables GAFmin and Psychosis are robust across Models I-III, regardless of whether rater variables (Model II) or center-specific variables (Model III) are added to the analysis. This robustness suggests that estimated effects of similar demand variables in other studies would have remained largely unchanged in response to the introduction of supply variables comparable to ours, but this is of course a conjecture. The estimated effect of the Suicidal variable, which is significant in Model I but not in Models II-IV, suggests that confounding might take place for this variable. This may partly be due to the apparent rater disagreement for this variable observed in Table 4. In Models II and III, the estimated effects of rater characteristics are quite similar concerning sign and magnitude, but the significance is clearly lower in the latter. This again suggest that the center-specific effects are not confounding the rater effects, but rather that the two groups of variables compete in regard to explanatory power.

Studies based on clinical data will typically not be able to include referral-specific effects, because different raters from different institutions seldom assess exactly the same patients. In our data, the referral-specific effects would possibly encompass referral information on sociodemographic factors (e.g., gender and age group), the need and availability for social support, and a detailed diagnosis classification system where we, by construction, allow a unique diagnosis for each referral. Comparing Model IV to the other models, we find relatively minor changes: The demand variables become less significant, which is not surprising because the referral-specific effects and the demand variables both describe the referrals/patients. Also for the rater variables, the results are mainly unchanged – except for rater experience which is significant in Model IV but not in Model III – and the standard deviation of the center-specific effects is equal in Models IV and III.

In a survey by Hermann et al., models based upon diagnostic- and socio-demographic information from administrative data sets are found on average to explain only 6.7% of the total variance, while more detailed sources of data on average explained 22.8% [17]. The comparison of explanatory power (adjusted R-squared) across data sets, or across transformations of the dependent variable has some limitations. With this caveat in mind, our Model IV explains 38.3% (Adj R-sq) of the total variation, which is relatively high compared to the studies surveyed by Hermann et al. Evidently, our inclusion of supply variables contributes to the relatively high share of total variation being explained. However, also the simpler models do relatively well in this respect; for instance Model I, which only includes demand variables, explains 16.6% of total variation in treatment costs.

Our models explain relatively high shares of the variation in log treatment costs, but much variation remains unexplained. The referrals, which are not standardized and quite often brief, leave room for interpretation and discretion to the assessors. When a treatment episode ends, the municipality assumes responsibility for the patient and, if necessary, provide living arrangements, social support etcetera. The availability of such after care resources may vary across catchment areas, and, if so, this again may influence the volume of treatment received at the centers. A survey of 139 mental health professionals familiar with inpatient settings showed a respondent’s belief in a patient’s symptomatology, level of adaptive functioning and social supports as more important length of stay determinants than diagnosis [12].

Some caution is required in drawing inferences about the impact of both demand and supply variables. First, our data is hypothetical and involve the clinicians’ assessments about future treatment. During actual treatment, the treating clinician gains information over time about the needs of each patient. It is possible that this will reduce the variability in treatment costs as compared to the results arrived at in our study. However, testing this hypothesis is not easy since, in a strict sense, comparable non-hypothetical patient data will not be available. Second, our results may not be representative for the whole country, because all 41 participating raters from 14 centers are from the South-East Health Region, covering 58% of the Norwegian population. Within the South-East Health region, all raters were invited to participate in the survey. The response rate was 60% and, thus, we cannot rule out the possibility of self-selection bias. Compared to the participants, the non-responders could potentially systematically have recommended different treatment costs or made different clinical assessments. Third, we have considered a relative limited number of referrals, and these referrals had identical weights (1/20) in the portfolio of referrals assessed by the clinicians. In actual clinical practice, the “patient types” represented by our referrals will have varying weights (relative frequencies), and additional patient types will clearly exist. Fourth, our study focuses on outpatients being treated at one particular type of institution (CMHCs), meaning that our findings may not be generalizable to other patient groups (e.g. inpatients) and other institutions (e.g. mental hospitals and psychiatric departments within somatic hospitals). Fifth, we do not consider indirect cost and potential variations in service organization [29, 31].

## Conclusions

For each referral the clinicians disagree substantially with respect to recommended treatment costs (a proxy for utilization), which seems unwarranted because the referrals are identical across clinicians. This within-referral variation suggests that clinicians differ both in their interpretation of needs and in their understanding of adequate treatment volumes for a given need. It implies a weakening of horizontal equity, because equal cases are not treated equally. It also points at a potential for economizing on scarce resources: because the cost distributions are right-skewed, reducing the variation could reduce average cost.

Our regression results confirm that supply variables (non-need variables) are important and that both institutional- and individual rater effects matter. The role identified for center-specific effects might reflect variations in organizational culture and resource availability (economics of scale, imperfectly risk adjusted budgets, input prices etc.); while ownership, teaching status, and payment structure are not plausible candidates since the participating centers (CMHCs) are homogenous in such respects. As concerning the individual rater variables, we find that both profession and managerial responsibility matter.

The role of supply variables (non-need variables) as significant explanatory variables for costs raises the question as to whether studies that rely on demand variables (need variables) produce biased coefficients. Our findings do not support this view for GAF and psychosis, while the coefficient for suicidality could be biased. Clearly, this conclusion is not a general one since the literature contains need variables beyond those accounted for in this study.

Future works should focus on providing insights into the particular mechanisms that lies behind the center effects. Furthermore, it would be of interest to investigate to what degree the introduction of standardized referral letters would reduce variability. It would also be of interest to see to what degree different theoretical orientations play a role for example by using social science methodologies such as interview data.

## Additional file

Additional file 1: Figure S1. Histogram of treatment costs and log treatment costs, with normal density plots; **Table S1.** Distribution of reccomended treatment types; **Table S2.** Estimates for fixed effects in models III and IV; **Table S3.** Estimates for fixed effects in models V-VII; **Table S4.** Variant of Models II and III, with rater-specific effects. (DOCX 42 kb)

## Abbreviations

CMHC: Community Mental Health Center
DRG: Diagnosis-Related Group
GAF-F: Global Assessment Scale – Functioning
GAF-S: Global Assessment Scale – Symptoms
GP: General Practitioner
NOK: Norwegian Kroner
OLS: Ordinary Least Squares

## References

[1] Tulloch AD, Fearon P, David AS (2011). Length of stay of general psychiatric inpatients in the United States: systematic review. Admin Pol Ment Health.

[2] McCrone P, Thornicraft G, Phelan M, Holloway F, Wykes T, Johnsen S (1998). Utilization and costs of community mental health services. PRiSM psychosis study. 5. Br J Psychiatry.

[3] Rupp A, Steinwachs DM, Salkver DS (1985). Hospital payment effects on acute inpatient care for mental disorders. Arch Gen Psychiatry.

[4] Frank RG, Lave JR (1986). The effect of benefit design on the length of stay of Medicaid psychiatric patients. J Hum Resour.

[5] Kirshner LA, Johnsen L (1985). Length of stay on a short-term unit. Gen Hosp Psychiatry.

[6] Freiman MP, Ellis RP, McGuire TG (1989). Provider response to Medicare’s PPS: reductions in length of stay for psychiatric patients treated in scatter beds. Inquiry.

[7] McCrone P, Phelan M (1994). Diagnosis and length of psychiatric in-patient stay. Psychol Med.

[8] Creed F, Tomenson B, Anthony P, Tramner M (1997). Predicting length of stay in psychiatry. Psychol Med.

[9] Ringard Å, Sagan A, Sperre Saunes I, Lindahl AK (2013). Norway: health system review. Health Syst Transit.

[10] Kato K, Igor I, Miner CR, Rosenblum JL (1995). Cognitive impairment in psychiatric patients and length of hospital stay. Compr Psychiatry.

[11] Caton CLM, Gralnick A (1987). A review of issues surrounding length of psychiatric hospitalization. Hosp Community Psychiatry.

[12] Mezzich JE, Sharfstein SS (1985). Severity of illness and diagnostic formulation. Classifying patients for prospective payment systems. Psychiatr Serv.

[13] Taube C, Lee ES, Forthofer RN (1984). Diagnosis-related groups for mental disorders, alcoholism, and drug abuse: evaluation and alternatives. Hosp Community Psychiatry.

[14] Horn SD, Chambers AF, Sharkey PD, Horn RA (1989). Psychiatric severity of illness: a case mix study. Med Care.

[15] Amaddeo F, Beecham J, Bonizzato P, Fenyou A, Tansella M, Knapp M (1998). The costs of community-based psychiatric care for first-ever patients: a case register study. Psychol Med.

[16] Bonizzato P, Amaddeo F, Chisholm D, Tansella M (2000). Community-based mental health care: to what extent are services costs associated with clinical, social and service history variable?. Psychol Med.

[17] Hermann RC, Rollins CK, Chan JA (2007). Risk-adjusting outcomes of mental health and substance-related care: a review of the literature. Harv Rev Psychiatry.

[18] Lave JR, Frank RG (1990). Effect of the structure of hospital payment on length of stay. Health Serv Res.

[19] Smith PC (2007). Formula funding of public services.

[20] Sutton M, Gravelle H, Morris S, Leyland A, Windmeijer F, Dibben C, Muirhead M. Allocation of resources to English areas: individual and small area determinants of morbidity and use of healthcare resources. Rep Dep Health 2002. http://www.nrac.scot.nhs.uk/wp-content/uploads/secure/docs/English%20allocation%20summary%20report%202002.pdf. Accessed 30 Aug 2017.

[21] Sutton M, Whittaker W, Morris S, Glover G, Dusheiko M, Wildman J, Gravell H, Burrows S, Simpson J, Fé-Rodríguez E, Birch S, Smith PC (2010). Report of the resource allocation for mental health and prescribing (RAMP) project: report to the Department of Health.

[22] Lyons JS, O’Mahooney MT, Larson DB (1991). The attending psychiatrist as predictor of length of stay. Hosp Community Psychiatry.

[23] Huntley DA, Cho DW, Christman J, Csernansky JG (1998). Predicting length of stay in an acute psychiatric hospital. Psychiatr Serv.

[24] Grepperud S, Holman PA, Wangen KR. Factors explaining priority setting at community mental health centres: a quantitative analysis of referral assessments. BMC Health Serv Res 2014; doi:10.1186/s12913-014-0620-3. 10.1186/s12913-014-0620-3 PMC4272526 25496562

[25] Jones SH, Thorinicroft G, Coffey M, Dunn G (1995). A brief mental health outcome scale-reliability and validity of the global assessment of functioning (GAF). Br J Psychiatry.

[26] Moos R, Nicol A, Moos B (2002). Global assessment of functioning ratings and the allocation and outcomes of mental health services. Psychiatr Serv.

[27] Tungström S, Söderberg P, Armelius B (2005). Special section on the GAF: relationship between the global assessment of functioning and other DSM axes in routine clinical work. Psychiatr Serv.

[28] Aas IHM. Guidelines for rating global assessment of functioning. Ann General Psychiatry 2011; doi:10.1186/1744-859X-10-2. 10.1186/1744-859X-10-2 PMC3036670 21251305

[29] Jones J, Amaddeo F, Barbui C, Tansella M (2007). Predicting costs of mental health care: a critical review. Psychol Med.

[30] Compton MT, Craw J, Rudisch BE (2006). Determinants of inpatient psychiatric length of stay in an urban county hospital. Psychiatr Q.

[31] Schneider J, Wooff D, Carpenter J, Brandon T, McNiven F (2002). Service organisation, service use and costs of community mental health care. J Ment Health Policy Econ.

