# Supplementary material for: Supply factors as determinants of treatment costs: clinicians’ assessments of a given set of referrals to community mental health centers in Norway
Source: BMC Health Serv Res. 2018 Jan 30;18:60. doi: 10.1186/s12913-018-2884-5 (PMC5789684; doi:10.1186/s12913-018-2884-5)
Supplement: Additional file 1: Figure S1. — Histogram of treatment costs and log treatment costs, with normal density plots; Table S1. Distribution of reccomended treatment types; Table S2. Estimates for fixed effects in models III and IV; Table S3. Estimates for fixed effects in models V-VII; Table S4. Variant of Models II and III, with rater-specific effects. (DOCX 42 kb) [file 12913_2018_2884_MOESM1_ESM.docx]

**Supplementary file**

**Figure S1. Histogram (density) of treatment costs and log treatment costs, with normal density plots (n=523)**

**Table S1. Distribution of reccomended treatment types^a)^**

| **Referral** | **N** | **Consultation** | | **Day-patient** | | **In-patient** | |
| --- | --- | --- | --- | --- | --- | --- | --- |
|  |  | **Mean** | **(n)** | **Mean** | **(n)** | **Mean** | **(n)** |
| 1 | 25 | 1.00 | (25) | 0.00 | (0) | 0.00 | (0) |
| 2 | 27 | 0.96 | (26) | 0.00 | (0) | 0.07 | (2) |
| 3 | 33 | 0.82 | (27) | 0.00 | (0) | 0.21 | (7) |
| 4 | 35 | 0.74 | (26) | 0.00 | (0) | 0.43 | (15) |
| 5 | 6 | 1.00 | (6) | 0.00 | (0) | 0.00 | (0) |
| 6 | 29 | 0.93 | (27) | 0.10 | (3) | 0.03 | (1) |
| 7 | 37 | 1.00 | (37) | 0.00 | (0) | 0.00 | (0) |
| 8 | 14 | 0.86 | (12) | 0.14 | (2) | 0.00 | (0) |
| 9 | 27 | 0.93 | (25) | 0.07 | (2) | 0.00 | (0) |
| 10 | 16 | 1.00 | (16) | 0.00 | (0) | 0.00 | (0) |
| 11 | 35 | 0.71 | (25) | 0.29 | (10) | 0.03 | (1) |
| 12 | 28 | 0.89 | (25) | 0.11 | (3) | 0.00 | (0) |
| 13 | 35 | 0.54 | (19) | 0.00 | (0) | 0.51 | (18) |
| 14 | 26 | 1.00 | (26) | 0.00 | (0) | 0.00 | (0) |
| 15 | 32 | 0.94 | (30) | 0.06 | (2) | 0.03 | (1) |
| 16 | 38 | 0.79 | (30) | 0.00 | (0) | 0.26 | (10) |
| 17 | 32 | 0.81 | (26) | 0.06 | (2) | 0.19 | (6) |
| 18 | 35 | 1.00 | (35) | 0.00 | (0) | 0.00 | (0) |
| 19 | 37 | 1.00 | (37) | 0.05 | (2) | 0.00 | (0) |
| 20 | 28 | 1.00 | (28) | 0.00 | (0) | 0.00 | (0) |
| Sum | 575 | 0.88 | (508) | 0.05 | (26) | 0.11 | (61) |

a) N = number of assessments; n = number of assessments that were assigned a treatment type (some clinicians assigned more than one treatment typer per referral); Mean = proportion of assessments that assigned a treatment type (e.g., for referral 2, consultations were assigned by 26 clinicians; Day-patient care was assigned by 0 clinicians; In-patient care was assigned by 2 clinicians).

**Supplements to Tables 5 and 6**

The base categories were chosen in order to make the average effect of the remaining dichotomous variable as close to zero as possible. The reported standard deviations of the estimated effects gives shorthand representations of the effects’ variation. For instance, when center C was used as reference in Model III, the full set of estimated center-specific effects is presented below. The average and standard deviation of these estimates are -0.013 and 0.280, respectively.

**Table S2. Estimates for fixed effects in models III and IV (Table 5) with robust standard errors**

|  | **Model III** | | | **Model IV** | | |
| --- | --- | --- | --- | --- | --- | --- |
|  | **Coeff** | **SE** | **p** | **Coeff** | **SE** | **p** |
| **Referral-specific fixed effects: 1** |  |  |  | 0.138 | 0.215 | 0.521 |
| **2** |  |  |  | 0.260 | 0.203 | 0.200 |
| **3** |  |  |  | 0.287 | 0.238 | 0.229 |
| **4** |  |  |  | 0.554 | 0.264 | 0.037 |
| **5** |  |  |  | -0.365 | 0.234 | 0.120 |
| **6** |  |  |  | -0.276 | 0.264 | 0.298 |
| **7** |  |  |  | 0.012 | 0.151 | 0.938 |
| **8** |  |  |  | -0.578 | 0.233 | 0.013 |
| **9** |  |  |  | -0.305 | 0.170 | 0.074 |
| **10** |  |  |  | -0.948 | 0.218 | <0.001 |
| **11** |  |  |  | 0.155 | 0.201 | 0.442 |
| **12** |  |  |  | -0.166 | 0.185 | 0.371 |
| **13** |  |  |  | 0.653 | 0.242 | 0.007 |
| **14** |  |  |  | 0.325 | 0.198 | 0.100 |
| **15** |  |  |  | 0.081 | 0.197 | 0.679 |
| **16** |  |  |  | -0.059 | 0.248 | 0.812 |
| **17** |  |  |  | 0.051 | 0.249 | 0.837 |
| **18** |  |  |  | -0.057 | 0.197 | 0.773 |
| **19** |  |  |  | -0.050 | 0.147 | 0.733 |
| **20 (base)** |  |  |  |  |  |  |
| **Center-specific fixed effects: A** | 0.176 | 0.197 | 0.374 | 0.092 | 0.199 | 0.643 |
| **B** | 0.132 | 0.165 | 0.423 | 0.107 | 0.162 | 0.511 |
| **C (base)** |  |  |  |  |  |  |
| **D** | -0.221 | 0.146 | 0.131 | -0.232 | 0.137 | 0.090 |
| **E** | 0.385 | 0.154 | 0.013 | 0.338 | 0.149 | 0.024 |
| **F** | -0.333 | 0.226 | 0.142 | -0.354 | 0.223 | 0.112 |
| **G** | -0.165 | 0.191 | 0.388 | -0.087 | 0.177 | 0.624 |
| **H** | -0.252 | 0.208 | 0.225 | -0.273 | 0.199 | 0.171 |
| **I** | -0.219 | 0.162 | 0.178 | -0.282 | 0.158 | 0.075 |
| **J** | 0.007 | 0.214 | 0.974 | -0.008 | 0.196 | 0.969 |
| **K** | 0.391 | 0.243 | 0.108 | 0.447 | 0.219 | 0.042 |
| **L** | 0.334 | 0.161 | 0.039 | 0.282 | 0.159 | 0.077 |
| **M** | -0.430 | 0.250 | 0.086 | -0.512 | 0.258 | 0.047 |
| **N** | 0.023 | 0.212 | 0.915 | 0.050 | 0.205 | 0.807 |

**Table S3. Estimates for fixed effects in models V-VII (Table 6) with robust standard errors**

|  | **Model V** | | | **Model VI** | | | **Model VII** | | |  |
| --- | --- | --- | --- | --- | --- | --- | --- | --- | --- | --- |
|  | **Coeff** | **SE** | **p** | **Coeff** | **SE** | **p** | **Coeff** | **SE** | **p** | |
| **Referall-specific fixed effects** 1 | 0.011 | 0.215 | 0.959 |  |  |  | 0.082 | 0.185 | 0.657 | |
| 2 | 0.161 | 0.192 | 0.402 |  |  |  | 0.278 | 0.173 | 0.108 | |
| 3 | 0.333 | 0.203 | 0.102 |  |  |  | 0.366 | 0.187 | 0.051 | |
| 4 | 0.846 | 0.188 | <0.001 |  |  |  | 0.899 | 0.172 | <0.001 | |
| 5 | -0.598 | 0.292 | 0.041 |  |  |  | -0.569 | 0.212 | 0.008 | |
| 6 | -0.281 | 0.227 | 0.218 |  |  |  | -0.258 | 0.215 | 0.230 | |
| 7 | -0.128 | 0.164 | 0.436 |  |  |  | -0.078 | 0.139 | 0.577 | |
| 8 | -0.472 | 0.241 | 0.051 |  |  |  | -0.485 | 0.220 | 0.028 | |
| 9 | -0.346 | 0.187 | 0.065 |  |  |  | -0.322 | 0.159 | 0.043 | |
| 10 | -1.134 | 0.222 | <0.001 |  |  |  | -0.964 | 0.195 | <0.001 | |
| 11 | 0.113 | 0.210 | 0.590 |  |  |  | 0.178 | 0.183 | 0.330 | |
| 12 | -0.239 | 0.189 | 0.205 |  |  |  | -0.218 | 0.176 | 0.214 | |
| 13 | 0.997 | 0.190 | <0.001 |  |  |  | 1.032 | 0.171 | <0.001 | |
| 14 | 0.161 | 0.191 | 0.399 |  |  |  | 0.216 | 0.163 | 0.185 | |
| 15 | 0.121 | 0.167 | 0.469 |  |  |  | 0.151 | 0.148 | 0.308 | |
| 16 | 0.278 | 0.184 | 0.132 |  |  |  | 0.311 | 0.154 | 0.044 | |
| 17 | 0.321 | 0.196 | 0.102 |  |  |  | 0.387 | 0.176 | 0.028 | |
| 18 | -0.189 | 0.183 | 0.302 |  |  |  | -0.152 | 0.157 | 0.332 | |
| 19 | -0.109 | 0.164 | 0.506 |  |  |  | -0.080 | 0.137 | 0.562 | |
| 20 (base) |  |  |  |  |  |  |  |  |  | |
| **Rater-specific fixed effects** 1  **f**  **Fixed effects effects: Raters** 1 |  |  |  | -0.128 | 0.228 | 0.574 | -0.096 | 0.155 | 0.536 | |
| 2 |  |  |  | 0.116 | 0.237 | 0.625 | 0.148 | 0.169 | 0.381 | |
| 3 |  |  |  | 0.287 | 0.264 | 0.276 | 0.322 | 0.190 | 0.091 | |
| 4 |  |  |  | 0.117 | 0.246 | 0.635 | 0.075 | 0.229 | 0.745 | |
| 5 |  |  |  | 0.735 | 0.203 | <0.001 | 0.684 | 0.179 | <0.001 | |
| 6 |  |  |  | 0.023 | 0.265 | 0.930 | -0.064 | 0.221 | 0.773 | |
| 7 |  |  |  | 0.048 | 0.217 | 0.824 | 0.130 | 0.207 | 0.533 | |
| 8 |  |  |  | 0.001 | 0.223 | 0.997 | 0.072 | 0.162 | 0.656 | |
| 9 |  |  |  | -0.241 | 0.261 | 0.357 | -0.169 | 0.161 | 0.296 | |
| 10 |  |  |  | -0.374 | 0.247 | 0.130 | -0.406 | 0.164 | 0.014 | |
| 11 |  |  |  | -0.383 | 0.271 | 0.159 | -0.364 | 0.168 | 0.031 | |
| 12 |  |  |  | -0.081 | 0.317 | 0.798 | -0.012 | 0.210 | 0.955 | |
| 13 |  |  |  | 0.328 | 0.205 | 0.111 | 0.452 | 0.137 | 0.001 | |
| 14 |  |  |  | 0.016 | 0.231 | 0.945 | 0.229 | 0.176 | 0.193 | |
| 15 |  |  |  | 0.539 | 0.165 | 0.001 | 0.574 | 0.132 | <0.001 | |
| 16 |  |  |  | 0.401 | 0.205 | 0.051 | 0.435 | 0.141 | 0.002 | |
| 17 |  |  |  | -0.099 | 0.271 | 0.715 | 0.041 | 0.174 | 0.812 | |
| 18 |  |  |  | 0.127 | 0.228 | 0.578 | 0.227 | 0.141 | 0.107 | |
| 19^a)^ |  |  |  | -0.088 | 0.216 | 0.685 |  |  |  | |
| 20 |  |  |  | -0.946 | 0.201 | <0.001 | -0.865 | 0.142 | <0.001 | |
| 21 |  |  |  | -0.353 | 0.276 | 0.201 | -0.256 | 0.229 | 0.264 | |
| 22 |  |  |  | -0.298 | 0.295 | 0.313 | -0.236 | 0.238 | 0.320 | |
| 23 |  |  |  | -0.218 | 0.263 | 0.406 | -0.246 | 0.183 | 0.180 | |
| 24 |  |  |  | -0.316 | 0.289 | 0.274 | -0.304 | 0.246 | 0.218 | |
| 25 |  |  |  | -0.187 | 0.277 | 0.499 | -0.190 | 0.215 | 0.376 | |
| 26 |  |  |  | 0.175 | 0.423 | 0.680 | 0.116 | 0.371 | 0.756 | |
| 27 |  |  |  | -0.181 | 0.205 | 0.377 | -0.140 | 0.171 | 0.413 | |
| 28 |  |  |  | -0.520 | 0.232 | 0.025 | -0.313 | 0.212 | 0.140 | |
| 29 |  |  |  | 0.141 | 0.259 | 0.587 | 0.210 | 0.191 | 0.272 | |
| 30 |  |  |  | 0.520 | 0.163 | 0.002 | 0.594 | 0.160 | <0.001 | |
| 31 |  |  |  | -0.167 | 0.188 | 0.375 | -0.121 | 0.276 | 0.662 | |
| 32 |  |  |  | -0.088 | 0.156 | 0.573 | 0.333 | 0.189 | 0.079 | |
| 33 |  |  |  | 0.356 | 0.226 | 0.116 | 0.258 | 0.160 | 0.107 | |
| 34 |  |  |  | 0.428 | 0.232 | 0.066 | 0.394 | 0.148 | 0.008 | |
| 35 |  |  |  | 0.581 | 0.189 | 0.002 | 0.627 | 0.130 | <0.001 | |
| 36 |  |  |  | 0.023 | 0.296 | 0.937 | -0.172 | 0.228 | 0.452 | |
| 37 |  |  |  | -0.175 | 0.282 | 0.536 | -0.292 | 0.236 | 0.216 | |
| 38 |  |  |  | -0.183 | 0.200 | 0.361 | -0.141 | 0.175 | 0.420 | |
| 39^b)^ |  |  |  |  |  |  | 0.072 | 0.166 | 0.665 | |
| 40 |  |  |  | -0.336 | 0.230 | 0.145 | -0.252 | 0.211 | 0.233 | |
| 41 |  |  |  | -0.504 | 0.222 | 0.023 | -0.398 | 0.171 | 0.021 | |
| Constant term | 11.386 | 0.146 | <0.001 | 11.495 | 0.156 | <0.001 | 11.404 | 0.177 | <0.001 | |

a) Model VI used rater 39 as base, while Model VII used rater 19 as base.

**Table S4. Variant of Models II and III, with rater-specific effects^a)^**

|  | **Coeff** | **SE** | **p** |
| --- | --- | --- | --- |
| **Constant term** | 11.777 | 0.305 | <0.001 |
| **GAFmin** | -0.016 | 0.004 | <0.001 |
| **Psychosis** | 0.486 | 0.091 | <0.001 |
| **Suicidal** | 0.030 | 0.071 | 0.670 |
| **Substance abuse** | -0.032 | 0.069 | 0.641 |
| **Rater-specific fixed effects 1**  **f**  **Fixed effects effects: Raters 1** | 0.045 | 0.262 | 0.865 |
| **2** | 0.418 | 0.260 | 0.108 |
| **3** | 0.543 | 0.286 | 0.058 |
| **4** | 0.374 | 0.297 | 0.209 |
| **5** | 1.015 | 0.249 | <0.001 |
| **6** | 0.200 | 0.274 | 0.467 |
| **7** | 0.393 | 0.271 | 0.147 |
| **8** | 0.223 | 0.256 | 0.385 |
| **9** | 0.009 | 0.275 | 0.975 |
| **10** | -0.038 | 0.251 | 0.881 |
| **11** | 0.003 | 0.281 | 0.991 |
| **12** | 0.037 | 0.296 | 0.901 |
| **13** | 0.697 | 0.250 | 0.006 |
| **14** | 0.690 | 0.327 | 0.035 |
| **15** | 0.945 | 0.229 | <0.001 |
| **16** | 0.774 | 0.243 | 0.002 |
| **17** | 0.208 | 0.278 | 0.453 |
| **18** | 0.425 | 0.248 | 0.088 |
| **19** | 0.108 | 0.248 | 0.662 |
| **20** |  |  |  |
| **21** |  |  |  |
| **22** | 0.002 | 0.320 | 0.994 |
| **23** | 0.021 | 0.270 | 0.939 |
| **24** | -0.031 | 0.329 | 0.925 |
| **25** | 0.048 | 0.291 | 0.868 |
| **26** | 0.497 | 0.411 | 0.227 |
| **27** | 0.126 | 0.249 | 0.612 |
| **28** | -0.020 | 0.302 | 0.947 |
| **29** | 0.646 | 0.286 | 0.024 |
| **30** | 0.853 | 0.241 | <0.001 |
| **31** | 0.038 | 0.444 | 0.931 |
| **32** | 0.386 | 0.225 | 0.086 |
| **33** |  |  |  |
| **34** | 0.610 | 0.237 | 0.010 |
| **35** | 0.664 | 0.245 | 0.007 |
| **36** | 0.931 | 0.231 | <0.001 |
| **37** |  |  |  |
| **38** | 0.017 | 0.287 | 0.953 |
| **39** |  |  |  |
| **40** | 0.352 | 0.257 | 0.171 |
| **41** | 0.080 | 0.278 | 0.774 |
| **Adj R-sq** | 0.277 |  |  |

a) 5 rater-specific effects were omitted due to multicollinearity. Adding rater background variables (*S_j_*) or center-specific effects (*u_ij_*) did not affect the estimated coefficients for clinical variables or adjusted R-squared, but it did increase the number of variables omitted due to multicollinearity.
